# *Hsa-miR-587* Regulates TGFβ/SMAD Signaling and Promotes Cell Cycle Progression

**DOI:** 10.22074/cellj.2020.6483

**Published:** 2019-10-14

**Authors:** Mahnaz Jahangirimoez, Abdallah Medlej, Mahmoud Tavallaie, Bahram Mohammad Soltani

**Affiliations:** 1. Department of Molecular Genetics, Faculty of Biological Sciences, Tarbiat Modares University, Tehran, Iran; 2. Department of Medical Genetics, Baqiyatallah University of Medical Sciences, Tehran, Iran

**Keywords:** Cancer, Cell Cycle, *miR-587*, TGFβ/SMAD Signaling

## Abstract

**Objective:**

Transforming growth factor beta/single mothers against decapentaplegic (TGFβ/SMAD) signaling pathway plays important roles in various biological processes. It acts as a tumor suppressor during the early stages of cancer progression. Discovering the regulators of this pathway provides important options for therapeutic strategies. Here, we searched for candidate microRNAs (miRNAs) that potentially target the critical components of the TGFβ signaling pathway.

**Materials and Methods:**

In the current experimental study, we first predicted miRNAs that target TGFβ components using a bioinformatics software. After that, quantitative real-time polymerase chain reaction (RT-qPCR) was used to detect the expression of miR-587, *TGFBR2, SMAD4, p21, CCND1* and *c-MYC* genes in transfected HEK293T and HCT116 cells. Dual Luciferase assay was performed to analyze the interactions between miRNAs and the target genes. Propidium iodide flow cytometry was used to determine cell cycle progression in HEK293T and HCT116 cells under hsa-miR-587 (miR-587) overexpression circumstances.

**Results:**

Multiple miRNA responsive elements (MREs) were predicted for *miR-587* within the 3’UTRs of the *TGFBR2* and SMAD4 genes. Overexpression of *miR-587* in HEK293T and HCT116 cells resulted in downregulation of *TGFBR2* and *SMAD4* genes. In addition, a downstream target gene of TGFβ/SMAD signaling, P21, was significantly downregulated in the HCT116 cells overexpressing miR-587. Dual luciferase assay analysis provided evidence that there is a direct interaction between *miR-587* and the 3’UTR sequences of *TGFBR2* and *SMAD4* genes. Moreover, miR-587 overexpression in HEK293T and HCT116 cells resulted in reducing the SubG1 cell populations in both cell lines, as detected by flow cytometry.

**Conclusion:**

Altogether, our data revealed an important role for *miR-587* in regulating TGFβ/SMAD signaling and promoting cell cycle progression. These characteristics suggest that *miR-587* is an important candidate for cancer therapy research.

## Introduction

Transforming growth factor beta/single mothers against decapentaplegic (TGFβ/SMAD) signaling plays crucial roles in various cellular processes, cell development, and carcinogenesis (1). TGFβ is a member of the superfamily of multifunctional cytokines that binds to the TGFBR1 and TGFBR2 receptor serine/threonine kinases. Binding of TGFβ to TGFBR1 and TGFBR2 leads to direct phosphorylation and activation of receptor-regulated SMAD (R-SMAD) proteins, SMAD2 and SMAD3. Activated R-SMADs form a heteromeric complex with the Co-SMAD protein, SMAD4. Once fully formed, the SMAD complex is translocated into the nucleus, where it associates with other transcriptional regulators to activate or suppress the transcription of specific target genes (2, 3). Although TGFβ signaling is known to induce apoptosis and cell cycle arrest during the early stages of carcinogenesis, it has been also shown to promote cancer progression and metastasis in the advanced stages of cancer (4, 5).

MicroRNAs (miRNAs) are small (18-24 nucleotides) non-coding RNAs that control gene expression posttranscriptionally (6). They are involved in the regulation of several biological processes. miRNAs generally bind to the 3¨@untranslated region (3¨@UTR) of their target mRNAs leading to their degradation or translational repression (7). Dysregulation of miRNA expression has been observed in various human tumors (8, 9). Interestingly, miRNAs are implicated in the process of carcinogenesis by acting as either tumor suppressors or oncogenes (10, 11). In addition, miRNAs play important roles in modulating signaling pathways by regulating the expression of their components. Various miRNAs have been reported to control core components of the TGF¦β/SMAD signaling pathways (12-16).

Here, we demonstrated that *miR-587* likely has a negative effect on the expression of TGFβ/SMAD signaling components. Bioinformatics analysis showed that *miR-587* has multiple recognition sites within the 3’UTRs of two essential components of the TGFβ pathway, the *TGFBR2* and SMAD4 genes. Overexpressing *miR-587* in HEK293T and HCT116 cells (TGFβ pathway-active cells) resulted in the downregulation of *TGFBR2* and *SMAD4* expression. Furthermore, dual luciferase assay results suggested a direct interaction between *miR-587* and the two target genes. Moreover, overexpression of *miR-587* resulted in reducing the SubG1-phase cellular population of HEK293T and HCT116 cells. The results of the current study suggest *miR-587* as an important regulator of TGFβ signaling pathway.

## Materials and Methods

### Bioinformatics tools

Prediction of miRNAs that target components of the TGFβ pathway was performed using the Targetscan (17), DIANA MicroT-CDS (18, 19) and miRmap (20) web servers. TargetScan predicts miRNA targets by searching for the presence of sites that match the seed region of each miRNA (17). DIANA MicroT-CDS has the potential to predict miRNA responsive elements (MREs) located in both the 3ˊ-UTRs and coding sequence (CDS) regions (18, 19). miRmap uses thermodynamic, evolutionary, probabilistic, or sequence-based features in its prediction process (20). The phylogenetic conservation of the predicted MREs of miR-587 within the 3´UTRs of its target genes was evaluated using the UCSC genome browser (21).

### Plasmid construction

The *miR-587* precursor was polymerase chain reaction (PCR)-amplified using a pair of specific primers (Table 1), and the PCR product was cloned into the multiple cloning site of the pmR-mCherry expression vector (Clontech, USA). The resulting construct was transformed and amplified into the DH5-Alpha *E.coli* bacterial strain and later extracted by mini-prep kit (Qiagen, Germany) and sequenced to verify the absence of any mutations.

### Cell culture

HEK293T and HCT116 cells were cultured in Dulbecco’s Modified Eagle Medium:Nutrient Mixture F-12 (DMEM/ F12) or RPMI media, respectively (Invitrogen, USA), supplemented with 100 U/ml penicillin, 100 μg/ml streptomycin (Sigma, USA), and 10% fetal bovine serum (Invitrogen, USA), and incubated at 37˚C with 5% CO_2_. HEK293T and HCT116 cells were obtained from Pasteur Institute (Tehran, Iran).

### Transfection

HEK293T or HCT116 cells were seeded in 12-well plates (12×10^4^ cells per well). Transfection was performed using Lipofectamine 2000 reagent according to the manufacturer’s instructions (Invitrogen, USA).

**Table 1:** Primers used in the study


Primer	Primer sequence (5ˊ-3ˊ)

Anchored Oligo dT	GCGTCGACTAGTACAACTCAAGGTTCTTCCAGTCACGACGT_18_N
*U48*	TGACCCCAGGTAACTCTGAGTGTGT
*miR-587*	GGCGCTTTCCATAGGTGATGAGT
Anchored reverse	GCGTCGACTAGTACAACTCAAG
*Pre-miR-587*	F: TCAGCTCAGACCACATTTCATCA
	R: ATGAGGACAGCCATGAGACAGAT
*GAPDH*	F: GCCACATCGCTCAGACAC
	R: GGCAACAATATCCACTTTACCAG
*CCND1*	F: CAGAGTGATCAAGTGTGACCC
	R: CGTCGGTGGGTGTGCAAGC
*c-MYC*	F: CTCCTACGTTGCGGTCACAC
	R: CGGGTCGCAGATGAAACTCT
*SMAD4-3ˊ*UTR	F: AAGTAATGGCTCTGGGTTGGG
	R: TCAAACAGCAGAACAAAGATAAGGAA
*TGFBR2-3ˊ*UTR	F: TTTGGATGGTGGAAGGTCTC
	R: GCAACAGCTATTGGGATGGT
*K-RAS-3ˊ*UTR	F: GTGAGGGAGATCCGACAATACAGA
	R: GCCGCGCTGCTGCTACCTTTGGGC


### RNA extraction, cDNA synthesis and quantitative real-time polymerase chain reaction

Total RNA was extracted using Trizol reagent according to the manufacturer’s instructions (Invitrogen, USA). Genomic DNA was removed by DNaseI treatment as the following: DNase I treatment (Takara, Japan) at 37˚C for 30 minutes, followed by heat and EDTA inactivation of the enzyme for 10 minutes. cDNA was synthesized using Prime Script II reverse transcriptase (Takara, Japan) according to the manufacturer’s instructions. For miRNA detection, polyA tailing was performed before cDNA synthesis using the E. coli Poly (A) Polymerase kit (NEB, England). Real-time PCR was performed according to standard protocols by StepOne™ system (Applied Biosystems, USA). *GAPDH* and *U48* expression levels were used to normalize the real-time PCR results.

### Western blot

Total cellular proteins were extracted from RiboXprecipitated cell extracts according to a recently reported protocol (22). The extracted protein concentrations were determined using Bradford assay (23). 40 ìg of each protein sample were separated by polyacrylamide gel electrophoresis and transferred to a polyvinylidene difluoride (PVDF) membrane. Primary antibodies against cyclin D1 protein (Santa Cruz, USA), β-actin (Santa Cruz, USA) and goat anti-mouse secondary antibody (BIORAD, USA) were diluted according to the manufacturers’ instructions. The expression levels of CCND1 protein was normalized against β-actin protein expression.

### Luciferase assay

The desired fragments of the 3ˊUTRs of *TGFBR2* and *SMAD4* genes in addition to a similar-sized fragment of the K-RAS_3ˊUTR (off target) were cloned downstream of the Renilla luciferase gene of the PSI-CHECK2 plasmid. The resulting constructs were co-transfected with the pmR-mCherry/pre-miRNA or pmR-mCherry vectors in HEK293T cells. 48 hours after transfection, dual luciferase assay was performed using Dual-Glo luciferase assay kit (Promega, USA).

### Cell cycle analysis

HEK293T or HCT116 cells transfected with miR- 587 or mock were collected 36 hours after transfection, centrifuged at 1200 rpm for 5 minutes and washed twice with phosphate buffered saline (PBS). Subsequently, cells were fixed in 1 ml of 70% ethanol for at least 30 minutes. For each sample, 500 μl propidium iodide staining solution was added to each sample and incubated for 30 minutes at room temperature. Cell cycle analysis was performed by the FACSCalibur flow cytometer (BD Biosciences, USA).

### Statistical analysis

The relative expression of the desired genes was calculated according to the 2^−ΔΔCt^ method. Real-time PCR results were normalized against the endogenous expression of the *U48* or *GAPDH* genes. GraphPad Prism 7 (GraphPad software, USA) was used to perform statistical tests (t test) and graph construction. Results with P<0.05 were considered statistically significant. All experiments were performed in triplicates.

## Results

### Bioinformatics analysis suggests miR-587 as an inhibitor of TGFβ signaling

**Fig 1:**
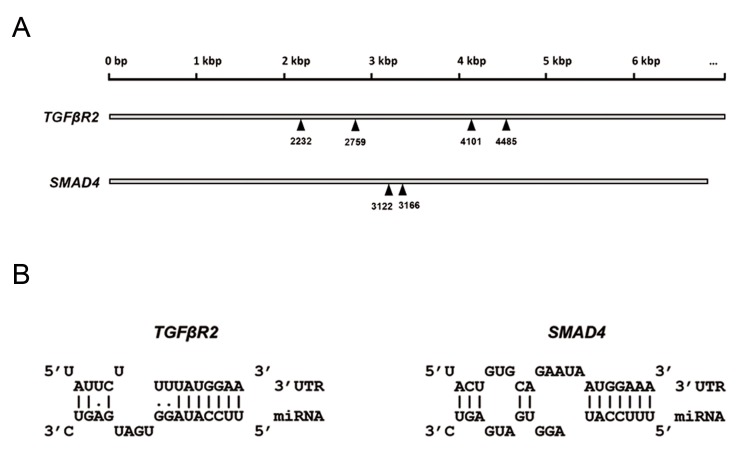
Predicted miRNA responsive elements (MREs) of *miR-587* within the 3ˊUTRs of *TFFBR2* and *SMAD4* genes. **A.** The positions of *miR-587* binding sites within the 3ˊUTRs of *TGFΒR2* and *SMAD4* transcripts. The numbers indicate the position of the first nucleotide of each MRE with respect to the transcription initiation site and **B.** Schematic representation of the base-pairing status between the *miR-587* seed sequence and the MREs of the target genes. One MRE is presented for each target gene.

A set of miRNAs was predicted by the utilized software to target important genes implicated in the TGFβ signaling pathway. For instance, hsa-let-7f-5p and hsa-miR-4458 were predicted to target TGFβR1, hsa-miR-302a-3p, hsa-miR-302d-3p and hsa-miR-587 were predicted to target TGFβR2 and hsa-miR-548g- 5p, hsa-miR-4288 and hsa-miR-587 were predicted to target the *SMAD4* gene. To select one of the candidate miRNAs for experimental validation, we focused on the miRNAs that can target more than one gene of the TGFβ signaling components at the same time. In addition, the scores obtained by the software used were taken into consideration and led to the final selection of *miR-587* as a potential candidate. In terms of its possible role, *miR-587* was predicted to potentially target two important genes related to the TGFβ signaling pathway, the *TGFBR2* and *SMAD4* genes (Fig .1A). Our bioinformatics predictions showed that each of the 3ˊUTRs of the *TGFBR2* and *SMAD4* genes contains more than one MRE for *miR-587*. Additionally, the seed sequence of *miR-587* showed high-scored base pairing with the targets’ MREs (Fig .1B).

### Downregulation of *TGFBR2* and *SMAD4* following the overexpression of *miR-587*

**Fig 2:**
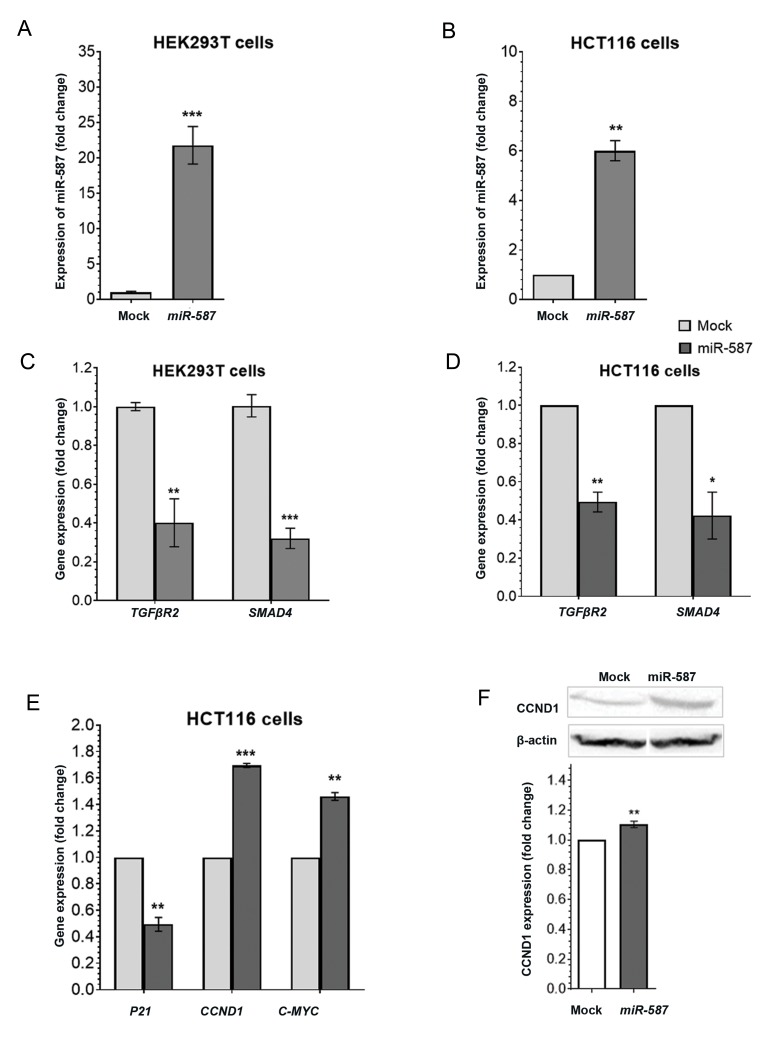
Effect of miR-587 overexpression on the expression of its predicted target genes. **A, B.** HEK293T and HCT116 cells transfected with the pmCherry/ pre-miR-587 construct or empty pmCherry control. Reverse transcription quantitative polymerase chain reaction (RT-qPCR) analysis indicated more than 25-fold overexpression of *miR-587* in HEK293T cells and ~7-fold in HCT116 cells following the transfection of the pmCherry/pre-miR-587 construct in comparison to controls (P=0.0002 and 0.0064, respectively), **C, D.** Following the overexpression of miR-587, the expression levels of *TGFBR2* and *SMAD4* reduced significantly in both HEK293T and HCT116 cell lines, **E.** Overexpressing of miR-587 in HCT116 cells resulted in the downregulation of p21 gene expression, and upregulation of *CCND1* and *c-MYC* genes, and **F.** Western blot analysis showed a significant upregulation in the CCND1 protein level in the HCT116 cells overexpressing miR-587 compared to control. RT-qPCR results were normalized according to the endogenous expression of *U48* and GAPDH genes. Western blot results were normalized according to the endogenous expression of β-actin protein. *; 0.01<P<0.1, **; P≤0.01 and ***; P≤0.001.

In order to validate the prediction results, *miR- 587* was overexpressed in HEK293T and HCT116 cells. Overexpression of *miR-587* was performed by transfecting the cells with the pmCherry/pre-miR-587 construct or empty pmCherry as a control. Significant overexpression of miR-587 was detected in both cell lines transfected by pre-*miR-587* in comparison to the controls (Fig .2A, B). As a result, RT-qPCR analysis indicated that *TGFBR2* and *SMAD4* expression levels decreased significantly in both cell lines (HEK293T and HCT116) overexpressing *miR-587* compared to the controls (Fig .2C, D). Moreover, the *P21* gene showed a significant downregulation in the HCT116 cells overexpressing *miR-587* compared to the controls, while *CCND1* and *c-MYC* genes showed a significant upregulation in the same samples (Fig .2E). In addition, western blot analysis confirmed these results and showed a significant upregulation of the CCND1 protein level in comparison to the control samples (Fig .2F).

### Interaction between *miR-587* and the 3ˊUTRs of the *TGFBR2* and *SMAD4* genes

**Fig 3:**
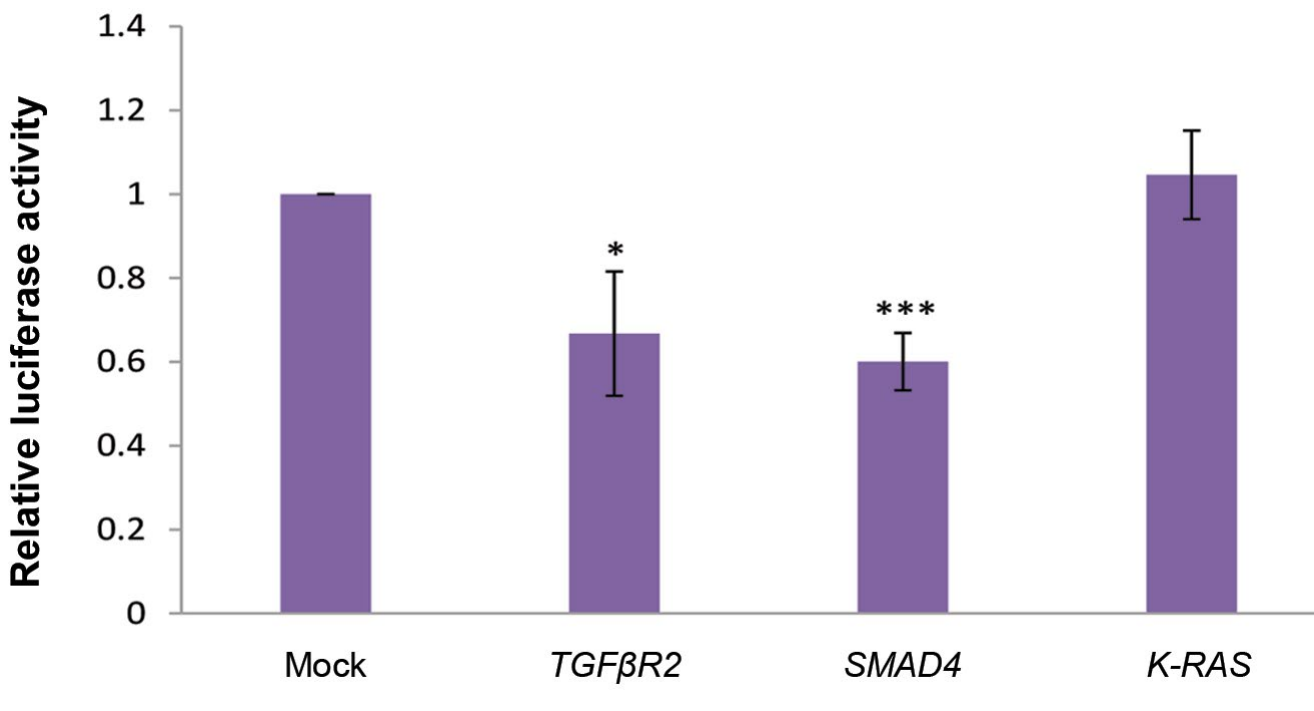
Direct interaction between *miR-587* and the 3ˊUTRs of *TGFBR2* and *SMAD4*. A significant luciferase activity reduction was detected in the HEK293T cells co-transfected with PSICHECK-2/*TGFBR2_3ˊ*UTR or PSICHECK-2/*SMAD4_3ˊ*UTR (P=0.02 and 0.0007, respectively), and pmCherry/pre-miR-587 construct, compared to mock or off-target (PSICHECK-2/*K-RAS_3ˊ*UTR) controls. *; P≤0.05 and ***; P≤0.001.

In order to examine a direct interaction between *miR- 587* and the 3ˊUTRs of *TGFBR2* and *SMAD4* genes, a dual luciferase assay was performed. HEK293T cells were co-transfected with PSI-CHECK2/TGFBR2_3ˊUTR, PSI-CHECK2/SMAD4_3ˊUTR or PSI-CHECK2/KRAS_ 3ˊUTR constructs and pmCherry/pre-*miR-587* or mock. PSI-CHECK2/K-RAS_3ˊUTR was used as an off-target. Dual luciferase analysis indicated a significant reduction in the luciferase activity of the cells co-transfected with miR-587 and its target genes 3ˊUTRs in comparison to the controls (Fig .3). These results suggest a direct interaction between *miR-587* and the 3ˊUTRs of *TGFBR2* and *SMAD4* genes.

### The effects of *miR-587* overexpression on cell cycle progression

**Fig 4:**
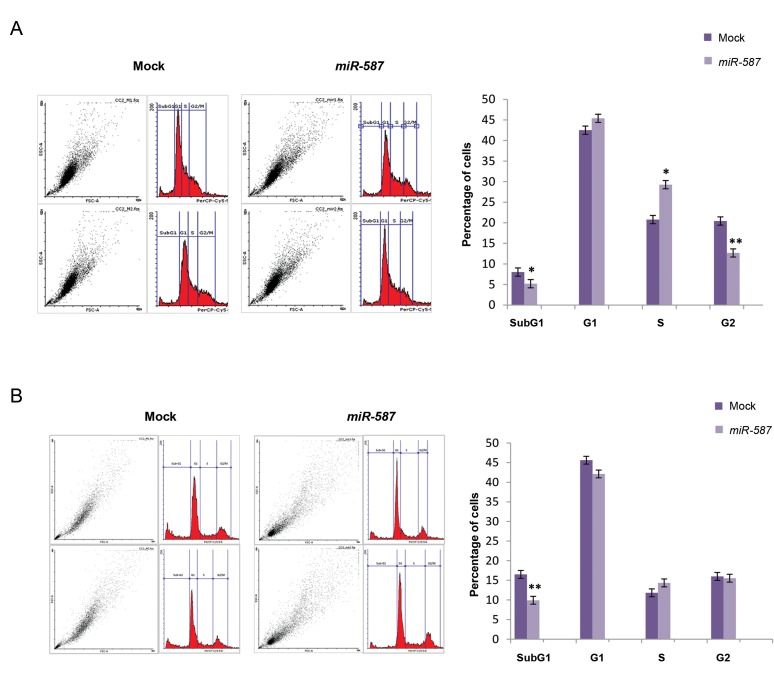
*Hsa-miR-587* overexpression effect on cell cycle status. **A.** Flow cytometry analysis indicated that the overexpression of *miR-587* in HEK293T cells resulted in a significant increase of the S-phase cell population (~1.5-folds, P=0.019), and a significant decrease in the SubG1-phase and G2-phase populations of cells (~1.5-folds, P=0.02 and 0.005, respectively) and **B.** Overexpression of *miR-587* in HCT116 cells resulted in a significant increase of the SubG1-phase cell population (~1.7-folds, P=0.01), but no significant variation was observed in the populations of the other cell cycle phases in comparison to the control. *; P≤0.05 and **; P≤0.01.

We used propidium iodide flow cytometry analysis to investigate the effects of *miR-587* overexpression on HEK293T and HCT116 cell cycle progression. The obtained results showed a significant reduction in the SubG1- phase and G2-phase populations of the HEK293T cells overexpressing *miR-587* compared to the controls (Fig .4A). In addition, a significant increase in the S-phase population of the HEK293T cells compared to the controls was observed (Fig .4). In HCT116 cells, on the other hand, overexpression of miR-587 resulted in a significant reduction of the SubG1- phase population of the cells, but no significant variations in cells in other cell cycle phases were observed in comparison to the controls (Fig .4B). These results suggest a role for *miR- 587* in arresting the progression of the cell cycle.

## Discussion

TGFβ/SMAD signaling pathway represents a complex network that effectively controls fundamental cellular processes. The activation of this pathway causes an arrest in the cell cycle of normal cells and early tumors. Mutational inactivation or dysregulated expression of the TGFβ/SMAD signaling components has been observed in human cancers (4, 24). TGFβ signaling has been shown to play a dual role in the course of cancer progression. It exhibits a tumor suppressive role in the early stages of the carcinogenesis process by inhibiting cell cycle progression and promoting apoptosis (5, 25). However, in the late stages, it exerts tumor-promoting effects by increasing tumor invasiveness and metastasis (4, 5).

Previous studies described TGFβ/SMAD signaling as an important inhibitor of cellular proliferation (26). TGFβ/SMAD signaling downregulates the expression of a set of genes resulting in the inhibition of cell cycle transition from G1 to S phase (27, 28). In the current study, in silico and experimental tools showed that miR- 587 targets important components of the TGFβ/SMAD signaling pathway. Moreover, overexpressing *miR-587* in HEK293T cells resulted in increasing the S-phase cell population, and reducing the SubG1-phase population. While in HCT116 cells, overexpressing this miRNA resulted in reducing the SubG1-phase only, without exerting any effect on the other cell cycle phases. This variation may be due to the differences between the two cell line origins, identities and status. However, the common effect exerted on the two cell lines at the SubG1- phase, indicates that miR-587 plays a role in the arrest of the cell cycle at this phase.

The dbDEMC 2.0 database that presents differentially expressed miRNAs in human cancers, has provided important evidence about the expression of *miR-587* in a set of human cancer samples (29). The available expression data showed that *miR-587* expression is upregulated in colon and breast tumors in comparison to normal tissue, and downregulated in high-grade colon tumors in comparison to low-grade tumors (5). These data provide an evidence about the function of this miRNA and its regulatory effects on the TGFβ signaling pathway that is downregulated in early tumor stages and upregulated during the late stages (30).

## Conclusion

In the current study, we demonstrated a regulatory role for *miR-587* against TGFβ/SMAD signaling. In addition, our results showed that *miR-587* promotes cell cycle progression.
